# Nurses` perspectives on early mobilization of intubated children in the pediatric intensive care unit: a qualitative study of barriers and facilitators

**DOI:** 10.3389/fped.2026.1853836

**Published:** 2026-06-03

**Authors:** Anne Grete Bjerkan, Marit Hulsund, Hilde B. H. Brenne, Mary-Elizabeth Eilertsen

**Affiliations:** 1 Children’s Clinic, Department of Pediatric Intensive Care, St.Olavs Hospital, Trondheim University Hospital, Trondheim, Norway; 2 Department of Public Health and Nursing, Faculty of Medicine and Health Sciences, Norwegian University of Science and Technology, Trondheim, Norway; 3 Children’s Clinic, Department of Neonatal Intensive Care Unit, St.Olavs Hospital, Trondheim University Hospital, Trondheim, Norway; 4 Pediatric Clinical Research Unit, St.Olavs Hospital, Trondheim University Hospital, Trondheim, Norway

**Keywords:** children, intensive care, interview, mobilization, nursing

## Abstract

**Purpose:**

To explore factors that promote or inhibit nurses’ early mobilization of intubated children, and to identify sources of reassurance for nurses during mobilization.

**Method:**

A qualitative study design was employed. Semi-structured interviews with eight specialist nurses working in a Norwegian Pediatric Intensive Care Unit (PICU) were analyzed using Braun and Clarke's reflexive thematic analysis.

**Results:**

Nurses described collegial support, clear communication, interdisciplinary collaboration, and shared understanding as key facilitators. Mobilization decisions were also shaped by the child's respiratory and hemodynamic stability and by available resources, physical space, and time. Nurses reported fear of causing harm and emphasized the need for further theoretical and practical training to increase confidence in mobilizing intubated children.

**Conclusion:**

Early mobilization was enabled by coordinated teamwork and interdisciplinary collaboration characterized by planning and communication, while barriers included time pressure, staffing constraints, limited space, inadequate equipment, perceived instability, and lack of physician endorsement. Strengthening nurses’ knowledge through clear guidelines and training like simulation may support safer and more consistent mobilization practices.

**Clinical implications:**

Future research should evaluate clinical outcomes of early mobilization in intubated children and assess how standardized protocols and training interventions influence implementation across diverse PICU settings.

## 1 Introduction

The first pediatric intensive care unit was established in Gothenburg in 1955, however intensive care for children was not recognized as a specialty until 1981 (1, 2). The establishment of the first pediatric intensive care units has contributed significantly to advances in both diagnostics and technology, resulting in a marked improvement in survival rates among critically ill children treated in these units (2–4).

Critically ill pediatric patients admitted to the PICU present with a wide range of life-threatening conditions, including multi-organ failure, severe infections such as sepsis or viral respiratory disease, traumatic injury, and complex congenital anomalies (5, 6). Some children are also admitted electively in the postoperative period following major surgical or diagnostic procedures (5, 6). In the PICU, many patients require invasive therapies and life-support measures, most common mechanical ventilation, in which a ventilator supports or replaces the child's spontaneous breathing (7–9). To tolerate these interventions, children typically receive sedatives and analgesics to ensure comfort, facilitate care, and prevent unintentional device removal such as self-extubating (9–11).

There has been a steady increase in the number of children admitted to PICUs globally over recent years, reflecting advances in pediatric care and increased survival of children with complex conditions (5, 6, 12–15).

With this increase in the number of children admitted to intensive care units and surviving intensive treatment, it is anticipated that more will experience long-term complications that can persist for years after discharge (16, 17). These complications are referred to as Post Intensive Care Syndrome (PICS), which encompasses the physical, cognitive, emotional, and social consequences of intensive care treatment for both the patient and the entire family (2, 16).

Previously, common practice involved deeply sedating children requiring mechanical ventilation through pain management and sedative medications (16). This approach leads to immobilization and sedation of the child during acute critical illness. Recent studies indicate that approximately three out of five children admitted to the PICU experience delirium, a sudden and temporary disturbance in attention, awareness and cognition during intensive care treatment (10).

Such an approach is now changing both nationally and internationally (9, 18). Clinical Practice Guidelines on Prevention and Management of Pain, Agitation, Neuromuscular Blockade and withdrawal (PANDEM) have been developed to ensure that risk- reducing measures become standard practice during PICU stays. Early mobilization is one of the interventions recommended in these guidelines (10).

Early mobilization aims to maintain and restore muscle and skeletal strength in critically ill children. It also helps minimize long-term complications of illness and intensive care treatment (9). A key area for improvement is ensuring that critically ill children remain as awake, active, and mobile as possible (2–4, 18). Balancing the need for activity and wakefulness in ventilated children may compromise comfort and increase stress during invasive mechanical ventilation. The incidence of patient- ventilator asynchrony is high during pediatric mechanical ventilation; studies indicate that children spend approximately one-third of their ventilatory time in asynchrony with the ventilator (7). Finding the optimal ventilator mode, while maintaining lighter levels of sedation and analgesia is challenging, yet crucial, especially when the goal is to enable early mobilization (7, 8).

Early mobilization has been associated with improved outcomes in critically ill patients and may reduce complications and length of stay; however, evidence in intubated children remains limited (2–4, 18). Research on children's complications of intensive care is limited, but studies on adults show that early mobilization leads to faster recovery compared to those who are not mobilized (9, 17–19). There is a need for further research into early mobilization of intubated children to improve outcomes after intensive care treatment and reduce the length of hospital stays (4, 16).

Mobilizing intubated children requires the presence of a multidisciplinary medical team. In Norway, nurses provide continuous bedside care, performing both essential nursing tasks and delegated medical procedures. During mobilization, an interdisciplinary team, typically physiotherapists, occupational therapists, and physicians, is required. These professionals participate in scheduled ward rounds, while the nurse remains the consistent coordinating figure throughout the patient's course of treatment.

Standardizing early mobilization for children in intensive care units is challenging due to the significant variations in age, size, diagnoses, and early mobilization is rarely systematized, with treatment often being random (9). For these reasons, there is a need to further examine the barriers, promote, and provide confidence for nurses during the mobilization process.

### 1.1 Aim of the study

The purpose of this study was to explore the nurses' experiences and perceptions regarding early mobilization of critically ill children who are intubated and receiving mechanical ventilation.

## 2 Materials and methods

### 2.1 Study design

The study has a qualitative design and Braun & Clarks reflexive thematic analysis were used to explore the interviews with specialized nurses working in a pediatric intensive care unit in Norway.

### 2.2 Recruitment

To ensure sufficient clinical experience with intubated children, inclusion in the study required a minimum of five years of experience working in the PICU and formal specialist education in pediatric or critical care nursing.

Of the 50 nurses employed in the pediatric intensive care unit, 42 met the inclusion criteria and were invited to participate, while eight were excluded due to not meeting the criteria (two lacked specialist nursing certification and six had less than five years of PICU experience).

Twelve nurses expressed interest in participating, and eight were ultimately included in the study; four did not proceed further or declined participation. Recruitment was discontinued when data saturation was reached, as no new themes or relevant information emerged from additional interviews.

Eligible nurses received a written invitation via email distributed by the head of the department. Nurses had between 8 and 19 years of experience working in the PICU; four were certified pediatric nurses and four were certified critical care nurses. None of the nurses held a master's or doctoral degree.

### 2.3 Study setting

The study participants worked in PICU, which has eight beds designated for high dependency and intensive care. In 2024, approximately 830 patients were admitted to this PICU, and of these, about 150 admissions lasting less than 24 h, 390 required high dependency care, and approximately 290 patients needed advanced intensive treatment. The facility also includes a five-bed recovery room, providing care to 2500–3000 pediatric patients annually. The department operates as a combined medical and surgical unit, delivering a broad spectrum of care to patients ranging in age from four weeks to approximately 16–18 years. The team includes around 50 nurses, anesthetists, and pediatricians who oversee daily clinical operations and provide on-call medical care.

### 2.4 Data collection

The data collection was conducted in September 2024 in a specific PICU. An interview guide with a semi-structured design was developed, and a pilot interview with the co-supervisor was conducted to evaluate the relevance and clarity of the questions in relation to the research's aims. Each participant took part in an individual interview. During the interviews, one of the authors acted as the interviewer, while the other supported the process by prompting when necessary and taking notes. Each interview lasted 30–45 min (Table1). Non-verbal communication was noted by the prompter. Participants were informed that they could get in touch afterward to add further information or elaborate on any topic. All interviews were audio recorded and automatically transcribed, and the transcripts were subsequently checked, anonymized and corrected verbatim by the authors before analysis.

**Table 1:** Interview guide.

Interview guide
Introduction Presentation Of The Interviewer And The Purpose Of The Study. Information About Anonymity, Confidentiality, And Voluntary Participation. Confirmation Of Informed Consent. Ask If The Participant Has Any Questions Before We Start. Background questions Can you tell a bit about your professional background and experience as a nurse? Experience with mobilizing intubated children How would you describe your experiences with mobilizing intubated children? How do you experience mobilizing intubated children? What do you need to feel safe in mobilization situations? What makes you choose to mobilize the child? What promotes mobilization for you? Can you give examples of mobilization situations that have been successful, and what contributed to that success? How do you experience collaboration with other healthcare professionals during mobilization situations? What contributes to good teamwork? In what way are relatives included in the child's mobilization situation? How is the child's autonomy safeguarded? If there is anything that inhibits you from mobilizing the child, what could it be? What do you consider the biggest challenges in mobilizing intubated children? Have you experienced situations where mobilization did not go as planned? What do you think was the reason for this? Practical considerations and strategies What techniques or strategies do you use when mobilizing intubated children? How do you assess the risks involved in mobilizing intubated children? Knowledge about mobilizing intubated children How do you perceive the level of knowledge in the department regarding mobilizing intubated children? What is the department as a whole lacking in order to mobilize all intubated children? What knowledge do you feel you are missing? Reflection and improvement How would you describe an ideal mobilization situation for intubated children? Is there anything you think could be improved regarding the mobilization of intubated children? Can you talk about a mobilization situation that made an impression on you? Conclusion Is there anything else you would like to add that we have not covered during the interview?

### 2.5 Qualitative data analysis and trustworthiness

**Figure 1:**
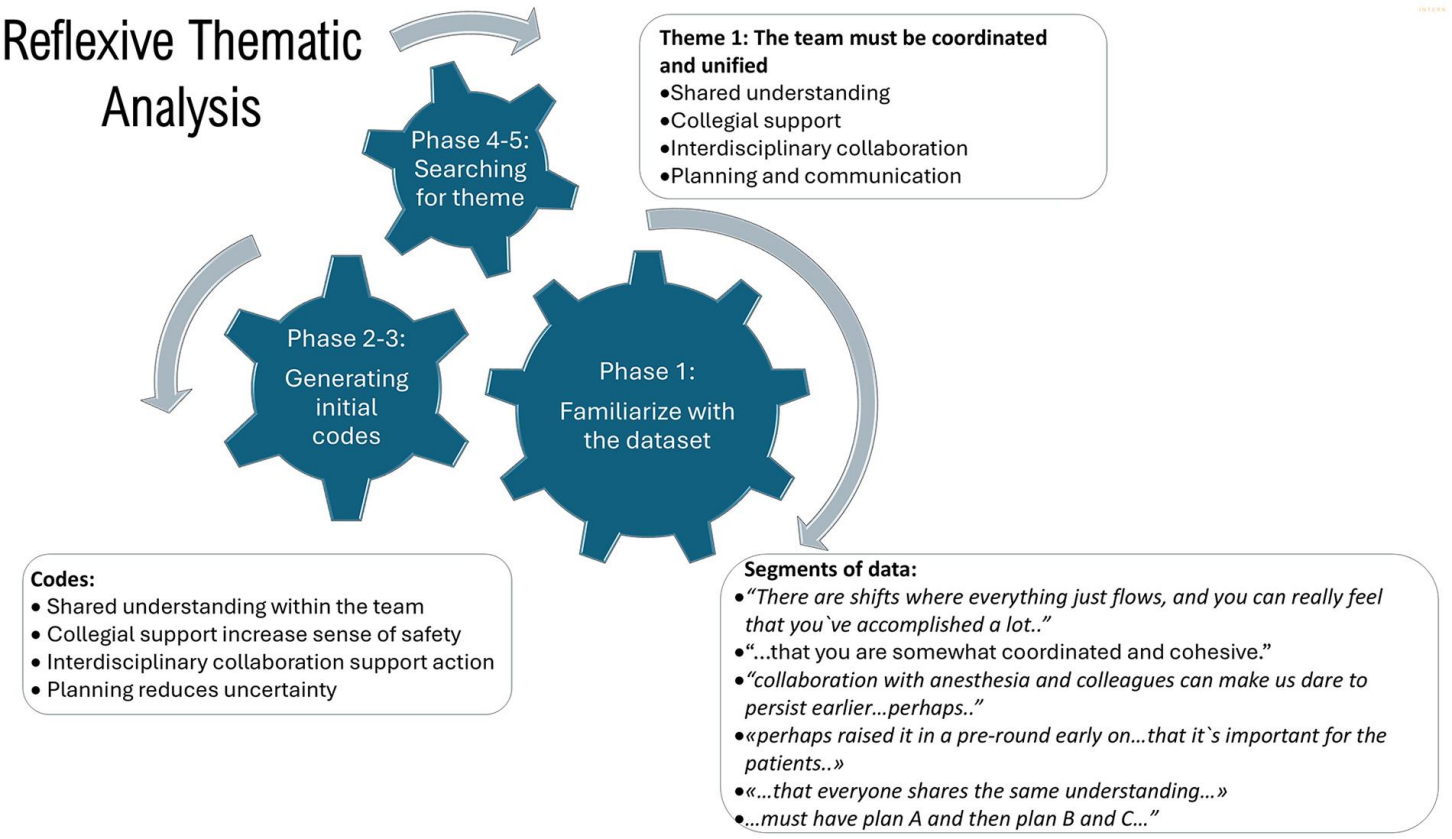
Illustation of the analysis.

Braun and Clarke's Reflexive Thematic Analysis (RTA) was used to analyze the data, an approach well suited to explore nurses`subjective experiences and perspectives. The analysis followed an iterative process that emphasizes the researcher`s active role in meaning making and moves from coding to the development and refinement of themes (20). RTA consists of six iterative phases. First, both first authors independently familiarized themselves with the dataset through repeated reading of the automatically transcribed texts, documenting preliminary interpretations of the participants`describtions. In the second phase, meaningful data segments were systematically coded in relation to the research questions. Codes were identified, compared, and iteratively refined through analytic dialogue among the first authors. In the third phase, codes were examined to identify broader patterns and potential themes. Initial themes were developed by organizing and clustering coded data to explore connections and overarching meanings. This interpretive process integrated data-driven insights with the first authors`analytic and theoretical understanding, resulting in nine preliminary themes. In the fourth phase, themes were reviewed independently and collaboratively to ensure coherence, clear boundaries, and accurate representation of the data. In the fifth phase, all authors collaboratively refined, defined, and named the themes, reviewing and selecting final data extracts that clearly and meaningfully represented each theme, resulting in four final themes (Figure 1). In the sixth and final phase, the analysis was compiled into a coherent analytic narrative, integrating the final themes with illustrative data extracts to present findings that meaningfully addressed the research questions (20).

### 2.6 Credibility

The researchers were colleagues of the participants, which may have influenced data generation through shared workplace norms, hierarchical dynamics, and social desirability. Although the researchers held no managerial authority over participants, their insider position may have affected what participants felt comfortable disclosing. Reflexivity was therefore maintained throughout the study to acknowledge and critically examine the researchers' roles and preunderstandings.

Several strategies were employed to enhance credibility. Participants were informed that participation was voluntary and confidential, with no workplace consequences.

Emphasis was placed on creating a relaxed and safe atmosphere, and the interviews were conducted in a meeting room outside the department. Interview questions explicitly addressed both facilitators and barriers to early mobilization to encourage critical reflection. During analysis, prolonged engagement with the data, reflexive memos and iterative discussions among the research team were used to challenge assumptions and support interpretations grounded in the data. The use of verbatim quotations further strengthened credibility by ensuring transparent linkage between participants' accounts and the analytic interpretations. Participant validation was not conducted; instead, credibility was supported through reflexive analytic memos, iterative team discussions, and close engagement with the dataset, consistent with reflexive thematic analysis.

### 2.7 Dependability

To ensure dependability, the analytic process followed a structured and transparent approach aligned with reflexive thematic analysis. All interviews were audio recorded and automatically transcribed, and the transcripts were subsequently checked, and corrected verbatim by the first authors prior to analysis. Decisions during coding and theme development were documented in reflexive analytic memos, providing a clear audit trail. Dependability was further supported through researcher triangulation, as the first authors worked both independently and collaboratively, using reflexive dialogue to examine and resolve differing interpretations and ensure analytic coherence rather than consensus.

### 2.8 Transferability

In qualitative research, where interviews are central, the results will inevitably be subjective, and reproducing the findings may be challenging (21). In this study, transferability is limited by its focus on a single Norwegian PICU with a small group of highly experienced female specialist nurses. To support analytic transferability, detailed descriptions of the study context, participants, and analytic process are provided, enabling readers to assess the relevance of the findings to other pediatric intensive care settings.

### 2.9 Authenticity

An effort was made to capture nurses' experiences and perspectives as authentically and accurately as possible. During the interviews, one of the authors systematically documented non-verbal expressions, including body language, facial expressions, pauses, and vocal cues, to enrich understanding of the nursè accounts.

## 3 Findings

The analysis generated four themes: (1) the team must be coordinated and unified, (2) resource requirements and priorities during mobilization, (3) pediatric intensive care, a delicate balance, and (4) knowledge provides confidence. Together, the themes show that early mobilization was experienced as a team-dependent and context-sensitive practice where perceived risk, available resources, and clinical confidence shaped whether and how mobilization was initiated.

### 3.1 The team must be coordinated and unified

The nurses described collegial support and a shared team mindset as central conditions for initiating mobilization. Some highlighted the significance of a unified understanding and good communication. A coordinated team was experienced as enabling both efficiency and a gentler approach to care, as illustrated by one nurse. These accounts suggest that coordination functions not only as a practical requirement but as a relational and cognitive condition that enables mobilization by reducing uncertainty and distributing responsibility within the team.

“There are shifts where everything just flows, and you feel that we accomplished a lot, were efficient while also being gentle in a way. Yes, that you are somewhat coordinated and cohesive.” (Nurse 7)

Interdisciplinary collaboration functioned as a key source of reassurance. Most nurses` emphasized that physician approval legitimized mobilization as safe and medically justified, and that ready access to the physician reduced uncertainty. At the same time, nurses highlighted that practical mobilization work was best supported by another nurse or a physiotherapist. One nurse summarized how collaboration increased willingness to act earlier:

“Collaboration with anesthesia and colleagues can somehow make us dare to persist earlier, perhaps.” (Nurse 3)

Planning emerged as a concrete strategy for managing uncertainty and shared responsibility. All nurses emphasized the importance of making a plan for mobilization and optimizing conditions to ensure everything went smoothly. This included having personnel and necessary equipment available. Pre-rounds were described as an important arena to align expectations and obtain a “go-ahead,” as Nurse 1 explained:

“That you have at least talked about it, and perhaps raised it in a pre-round early on, that this is something important for the patients, and that we get the go-ahead that it is okay for us to do.” (Nurse 1)

Several nurses stressed that a robust plan should include contingencies:

“Yes, things often do not go as planned, but then you have to make a new plan. When mobilizing, you must have plan A, and then plan B and C.” (Nurse 8)

### 3.2 Resource requirements and priorities during mobilization

Early mobilization was consistently described as resource intensive and dependent on staffing, time, and equipment. Nurses` framed resources primarily as a safety prerequisite:

“We need to have the resources ourselves so that it is safe to carry out.” (Nurse 5)

Resource constraints shaped prioritization and sometimes resulted in mobilization being postponed or omitted. Time pressure was frequently described as the most immediate barrier:

“If you have time, right? That hinders me. Maybe it's very busy, lots of things to do, and you don't feel you can prioritize it.” (Nurse 7)

Nurses also noted that competing demands could influence sedation practices in multi-bed room, as one nurse described:

“If there is a lot going on the other side of the room, it might not be possible to do it that day. Maybe you actually have to sedate the child more because so much is happening on the other side.” (Nurse 8)

These accounts suggest that constrained conditions can indirectly affect readiness for mobilization by limiting attention and increasing the perceived need to control the environment.

The physical environment and access to appropriate equipment were described as practical determinants of feasibility. While some nurses emphasized limited space as a clear barrier, others highlighted workarounds and adaptation:

“We manage, but looking from the outside, we have poor spatial conditions. The physical environment is not very accommodating in that regard, but we are creative, in a way.” (Nurse 7)

In addition, several described insufficient knowledge about available assistive devices as limiting the ability to mobilize efficiently and safely.

### 3.3 Pediatric intensive care, a delicate balance

Nurses` described mobilization decisions as contingent on ongoing assessments of respiratory and hemodynamic stability. Several noted that even small movements could trigger instability in the most critically ill children, leading to avoidance of mobilization:

“I experience that early mobilization can be difficult..and many times, with our most critical ill patients, we have noticed that even just moving the body a little causes volume change in the ventilator and blood pressure fluctuations. So, then it's like..we don't do anything.” (Nurse 3)

Sedation and pain management were viewed as key conditions for a mobilization attempt to be both tolerable and safe. Nurses described the challenge of maintaining an “optimal” level that avoids both over-sedation and excessive distress:

“ I think the challenge is actually doing it in such a way that you manage to keep the tube and maintain it at an appropriate sedation level, so they’re not too sleepy but also not too uncooperative. It's a fine balance.” (Nurse 4)

These accounts indicate that mobilization was not perceived as a discrete intervention, but as embedded in continuous clinical balancing between comfort, cooperation, and physiological stability.

These accounts further suggest that mobilization can be understood as a continuous balancing process between stability, comfort, and participation, reflecting a dynamic process of clinical judgement rather than a discrete intervention.

While most accounts focused on clinical stability, one nurse particularly emphasized communication and preparation as contributors to successful mobilization, highlighting variation in what was foregrounded as “key” to success. Although nurses largely agreed on the importance of safety, what they considered most critical varied, with some emphasizing clinical stability, others communication and planning, and others relational support such as caregiver presence. Several also described caregivers as a stabilizing presence for the child during mobilization:

“I have relatives standing in front, so the child has something familiar to look at. Then they are the security the child sees.” (Nurse 8)

### 3.4 Knowledge provides confidence

Fear was reported by all nurses as a central barrier that constrained early mobilization. Nurses described fear of tube dislodgement, causing pain or harm, and triggering instability. Importantly, this fear persisted despite most nurses reporting no direct experience with serious adverse events during mobilization. Nurse 2 captured this tension:

“It's this fear of losing equipment that I’m most afraid of. Because I’m so scared of doing something harmful or making it worse..I don't think I have ever been involved in an accidental extubating.” (Nurse 2)

This pattern may be described as a “fear–experience gap,” where perceived risk remains high despite limited prior experience of adverse events. This gap suggests that emotional risk perception continues to influence decision-making even when serious adverse events are rare.

Nurses also described knowledge as an enabling factor that could counterbalance uncertainty. Several requested more theoretical understanding of mechanisms and outcomes

“We need more theoretical knowledge about what it does to the body, the healing process, and to progress out of intensive care.” (Nurse 2)

Others emphasized a need to understand whether mobilization makes a difference for ventilator duration and hospital stay:

“..we should know more..if there's something we can do to promote shorter time on the ventilator and overall hospital stay. Whether early mobilization makes a difference.” (Nurse 6)

Practical training and clearer procedural guidance were repeatedly requested. Some nurses highlighted the absence of a protocol with inclusion/exclusion criteria and suggested that guidelines could support more independent mobilization decisions:

“If we had a protocol or guideline, you could take mobilization and the decision to mobilize more independently.” (Nurse 2)

Several also described variation in competence within the unit, highlighting that confidence develops through knowledge and access to clear reference points for decision-making:

“Very dependent on confidence, I would say..and I think that comes with gaining more knowledge and having some fixed points to relate to.” (Nurse 2)

## 4 Discussion

Early mobilization of intubated children emerged as a context-dependent decision practice shaped by the interaction of organizational conditions, interdisciplinary collaboration, and emotional–cognitive factors. These findings align with previous PICU litterature emphasizing the importance of both structural readiness and unit culture (9, 16, 17, 22). However, as this study did not assess clinical outcomes, any links between mobilization and outcomes should be interpreted as implications supported by prior evidence rather than findings of the present study (9, 14, 16–19, 22).

### 4.1 The team must be coordinated and unified

The findings indicate that team coordination functions not only as a practical facilitator but also as a safety mechanism that reduces uncertainty and distributes responsibility during high-risk care. Nurses emphasized that trusted collegial support and a shared understanding of early mobilization increased their sense of security, whereas reduced trust increased stress and perceived responsibility. This resonates with research suggesting that shared attitudes and unit culture influence confidence and sustainability of mobilization practices (22, 23). Our findings suggest that interdisciplinary collaboration transforms mobilization from an individual risk into a shared clinical decision. We interpret this as a process where responsibility is distributed across the team, which may increase confidence and reduce perceived risk. This is consistent with previous PICU research highlighting the importance of interprofessional teamwork for successful mobilization (16, 17, 22).

A notable pattern in our findings was that participants sought physician approval to confirm that mobilization was medically justified, but preferred practical assistance from nurses or physiotherapists during execution. Similar dynamics have been described in PICU settings, where medical endorsement may enable initiation while nursing and physiotherapy competence supports safe enactment (14, 17, 22). From an implementation perspective, this suggests that strengthening mobilization practice may require both clear medical decision frameworks and available practical support. Increased physiotherapy involvement has been highlighted as a valuable resource for cultivating early mobility culture in pediatric intensive care (9, 16, 17).

Nurses described planning and communication as key strategies for managing uncertainty, particularly through pre-round discussions and contingency planning. We interpret this as a form of anticipatory risk management, where preparation enables more confident and coordinated decision-making. This interpretation aligns with existing recommendations emphasizing individualized mobilization planning tailored to the child's condition and developmental level. In addition, nurses' emphasis on planning intersects with evidence suggesting that caregiver involvement may further support mobilization feasibility and child comfort in PICU settings (14, 24, 25).

### 4.2 Resource requirements and priorities during mobilization

Consistent with previous research, participants described time pressure, staffing shortages, and competing clinical tasks as major barriers to early mobilization in intensive care settings (3, 16, 17, 22). Importantly, our findings suggest that resources are experienced primarily as conditions for safe practice, rather than optional support. When staffing, equipment, or time were insufficient, mobilization was more likely to be deprioritized, and the threshold for perceived “acceptable risk” increased. This mirrors broader ICU literature identifying workload and resource scarcity as structural constraints that limit early mobility implementation (3, 16, 22).

Limited resources and time pressure may also force nurses to prioritize tasks, sometimes at the expense of ethical considerations. This highlights how resource constraints not only affect feasibility but may also contribute to moral tension in clinical decision-making

Physical space and access to age-appropriate equipment were also highlighted as barriers, consistent with previous PICU work (3, 16, 17, 22). Although technological tools have been developed to support pediatric mobilization, like adapted walkers, chairs, interactive tools, the literature also notes challenges related to cost, variable evidence, and availability (9, 16, 19). In practice, this suggests that implementation should prioritize feasible and scalable equipment, along with staff competence in using available assistive devices, rather than relying on expensive technologies with limited uptake (16).

In addition, the broader healthcare context may intensify these barriers. System pressures such as demographic changes, increasing prevalence of chronic conditions, workforce shortages, and capacity constraints are expected to challenge the sustainability of healthcare services in Norway (15, 26). However, this study did not quantify resource use or costs; therefore, claims regarding economic benefits should be interpreted as potential implications supported by previous research rather than findings from the present study (3, 9).

### 4.3 Pediatric intensive care, a delicate balance

Nurses described early mobilization of intubated children as a “delicate balance,” reflecting continuous negotiation between potential benefits and perceived risk. Hemodynamic and respiratory instability were consistently described as major constraints, particularly in the most critically ill children, and even minor movement could trigger physiological changes that led nurses to refrain from mobilization (9, 16, 27, 28).

Importantly, our findings suggest that “stability” operates not only as a physiological criterion but also as a confidence threshold shaped by uncertainty, prior experiences, and perceived support. When instability was anticipated, nurses described a tendency to avoid mobilization, which aligns with literature identifying instability as a key barrier to early mobility in PICU contexts (9, 27, 28).

The “delicate balance” described by nurses also reflects an ethical tension between beneficence and non-maleficence (29). While mobilization may promote recovery, nurses emphasized that the possibility of harm, such as accidental extubation or pain, must be weighed carefully against potential benefit, particularly in intubated children (21, 30).

Sedation and pain management were described as prerequisites for feasible mobilization, echoing recommendations emphasizing comfort, appropriate sedation strategies, and systematic monitoring to support safe mobilization and prevent distress (10, 11, 22). Nurses' emphasis on balancing “too sedated” vs. “too uncooperative” reflects the complexity of maintaining comfort while enabling activity. This aligns with evidence that delirium and withdrawal are common and require developmentally appropriate prevention and monitoring strategies, supported by structured assessment tools (10, 11). Because patient-ventilator interactions can influence comfort and tolerance, literature emphasizing ventilator synchrony and minimizing distress provides additional clinical context, yet these points should be interpreted as supportive background rather than as directly evidenced by our qualitative dataset (7, 8).

Finally, caregiver involvement emerged as an important relational condition that may support mobilization feasibility. Nurses described caregiver presence as reassuring for the child and supportive during mobilization, consistent with Family-Centered Care (FCC), a well-established approach in PICU in which caregiver involvement is associated with reduced stress and improved comfort for both children and families (14, 24, 25). In our study, caregiver presence appeared to function not only as emotional support, but also as a facilitating factor for mobilization, suggesting that relational factors may enhance both feasibility and perceived safety.

Overall, these findings suggest that early mobilization in PICU is shaped by dynamic interactions between physiological readiness and relational and organizational conditions, including preparation, familiarity, caregiver presence, interdisciplinary endorsement, which together influence bedside decision-making.

### 4.4 Knowledge provides confidence

A central finding was that emotional fear, particularly fear of tube dislodgement, instability, and causing harm, was described as a major barrier to early mobilization. These concerns align with patient safety considerations and nurses' ethical responsibility to prevent harm and suffering (28, 30). Notably, this fear persisted despite limited experience of serious adverse events. Evidence from large-scale studies indicates that unintentional extubation during mobilization is rare (14), yet nurses still described considerable hesitation. This pattern reflects a “fear–experience gap,” suggesting that perceived risk may continue to influence decision-making even when actual event frequency is low.

Nurses also emphasized knowledge and competence as key enablers that may counterbalance this uncertainty. They described a need for both theoretical understanding of potential benefits and underlying mechanisms, and practical training to support safe mobilization, including handling equipment and assistive devices. Several nurses also highlighted the absence of clear guidelines, including inclusion and exclusion criteria, and suggested that such tools could support more consistent and independent decision-making. These findings align with previous research indicating that standardized protocols and structured approaches can increase mobilization frequency and support implementation in pediatric intensive care settings (14, 22, 27). Simulation-based training has also been proposed as a strategy to enhance nurses' self-efficacy and clinical competence in early mobility practice (31).

Together, these findings suggest that knowledge functions as a mediator between fear and action. Confidence appears to be strengthened not only through access to information, but through practical rehearsal, clear decision criteria, and predictable routines that reduce uncertainty (14, 22, 31). In this way, competence may transform perceived risk into manageable clinical judgement, facilitating more confident and consistent mobilization practices.

Overall, the findings suggest that early mobilization of intubated children is influenced by an interdependent set of organizational, professional, and emotional factors. Implementation strategies may therefore be most effective when these factors are addressed together, including ensuring feasible resources, strengthening interdisciplinary coordination and physician endorsement, and building competence through protocols and simulation-based training (16, 17, 22, 27, 31). As this study did not assess outcomes such as adverse events, ventilation duration, length of stay, or costs, potential benefits should be interpreted as implications supported by prior literature rather than as findings from the present study (9, 14, 16–19, 22). Future research should therefore evaluate clinical outcomes and examine how guideline development and competence-building interventions influence implementation across diverse PICU contexts (27, 28).

## 5 Strengths and limitations

In line with the principles of RTA, continuous reflection was maintained throughout the process regarding how the relationship between the participants may have influenced the research, as well as how the researchers' own preunderstanding may have affected the study. As colleagues of the nurses, we recognized the risk of bias and took steps to minimize its impact. We emphasized voluntary participation, ensured confidentiality, and reflected continuously on how our dual roles might influence the research process. By acknowledging these dynamics and being transparent in our reporting, we aimed to uphold ethical standards and the trustworthiness of our findings. This relationship may have influenced the findings; however, it can also be considered a strength in terms of their preunderstanding of the data material. Within RTA, the researchers' personal positioning and preunderstanding are acknowledged as integral to shaping the analytical process.

It may be considered a limitation that all participants were female nurses with extensive experience; however, this reflects the actual gender distribution and level of experience among nurses in the unit. The interviews were conducted at a single pediatric intensive care unit in Norway, which may also be viewed as a limitation of the study. Therefore, the findings cannot be generalized to all intensive care units with different structures and staffing. Nevertheless, due to the study's methodological rigor and transparency, the findings may be considered transferable to similar units with comparable organization and structure.

## 6 Conclusions

Early mobilization of intubated children is shaped by an interdependent set of organizational, professional, and emotional factors. While collegial support, shared understanding, and interdisciplinary collaboration facilitate mobilization, constraints such as limited resources, clinical instability, and fear of harm contribute to cautious decision-making.

The findings highlight that knowledge and competence play a central role in mediating between fear and action, underscoring the importance of clear guidelines, structured planning, and competence-building strategies such as simulation-based training.

As this study did not assess clinical outcomes, potential benefits should be interpreted as implications supported by prior literature. Future research should therefore examine clinical outcomes and evaluate how standardized protocols and training interventions influence implementation in PICU settings.

## 7 Recommendations for further research

Research into nurses' experiences with early mobilization of intubated children highlights the need for clearer guidelines and structured approaches in pediatric intensive care. Establishing standardized routines may enhance nurses’ knowledge and support more consistent practice across units.

Previous studies suggest that early mobilization may be associated with reduced complications, improved patient and family experiences, and shorter hospital stays; however, these potential benefits are derived from external evidence and were not assessed in the present study.

Future research should therefore focus on clinical outcomes, including late complications and length of hospital stay, as well as the implementation and impact of standardized protocols in diverse PICU settings.

## Data Availability

The raw data supporting the conclusions of this article will be made available by the authors, without undue reservation.
